# SARS-CoV-2 serology increases diagnostic accuracy in CT-suspected, PCR-negative COVID-19 patients during pandemic

**DOI:** 10.1186/s12931-021-01717-9

**Published:** 2021-04-23

**Authors:** Jochen Schneider, Hrvoje Mijočević, Kurt Ulm, Bernhard Ulm, Simon Weidlich, Silvia Würstle, Kathrin Rothe, Matthias Treiber, Roman Iakoubov, Ulrich Mayr, Tobias Lahmer, Sebastian Rasch, Alexander Herner, Egon Burian, Fabian Lohöfer, Rickmer Braren, Marcus R. Makowski, Roland M. Schmid, Ulrike Protzer, Christoph Spinner, Fabian Geisler

**Affiliations:** 1 Department of Internal Medicine II, School of Medicine, Technical University of Munich, Munich, Germany; 2 German Center for Infection Research (DZIF), partner site Munich, Munich, Germany; 3 Institute for Virology, School of Medicine, Technical University of Munich, Munich, Germany; 4 Institute for Medical Statistics and Epidemiology, School of Medicine, Technical University of Munich, Munich, Germany; 5 Department of Anaesthesiology and Intensive Care Medicine, School of Medicine, Technical University of Munich, Munich, Germany; 6 Institute for Medical Microbiology, Immunology and Hygiene, School of Medicine, Technical University of Munich, Munich, Germany; 7 Institute for Diagnostic and Interventional Radiology, School of Medicine, Technical University of Munich, Munich, Germany

**Keywords:** COVID-19, SARS-CoV-2, Serology, Computed tomography, Efficacy, Accuracy

## Abstract

**Background:**

In the absence of PCR detection of SARS-CoV-2 RNA, accurate diagnosis of COVID-19 is challenging. Low-dose computed tomography (CT) detects pulmonary infiltrates with high sensitivity, but findings may be non-specific. This study assesses the diagnostic value of SARS-CoV-2 serology for patients with distinct CT features but negative PCR.

**Methods:**

IgM/IgG chemiluminescent immunoassay was performed for 107 patients with confirmed (group A: PCR + ; CT ±) and 46 patients with suspected (group B: repetitive PCR-; CT +) COVID-19, admitted to a German university hospital during the pandemic’s first wave. A standardized, in-house CT classification of radiological signs of a viral pneumonia was used to assess the probability of COVID-19.

**Results:**

Seroconversion rates (SR) determined on day 5, 10, 15, 20 and 25 after symptom onset (SO) were 8%, 25%, 65%, 76% and 91% for group A, and 0%, 10%, 19%, 37% and 46% for group B, respectively; (p < 0.01). Compared to hospitalized patients with a non-complicated course (non-ICU patients), seroconversion tended to occur at lower frequency and delayed in patients on intensive care units. SR of patients with CT findings classified as high certainty for COVID-19 were 8%, 22%, 68%, 79% and 93% in group A, compared with 0%, 15%, 28%, 50% and 50% in group B (p < 0.01). SARS-CoV-2 serology established a definite diagnosis in 12/46 group B patients. In 88% (8/9) of patients with negative serology > 14 days after symptom onset (group B), clinico-radiological consensus reassessment revealed probable diagnoses other than COVID-19. Sensitivity of SARS-CoV-2 serology was superior to PCR > 17d after symptom onset.

**Conclusions:**

Approximately one-third of patients with distinct COVID-19 CT findings are tested negative for SARS-CoV-2 RNA by PCR rendering correct diagnosis difficult. Implementation of SARS-CoV-2 serology testing alongside current CT/PCR-based diagnostic algorithms improves discrimination between COVID-19-related and non-related pulmonary infiltrates in PCR negative patients. However, sensitivity of SARS-CoV-2 serology strongly depends on the time of testing and becomes superior to PCR after the 2^nd^ week following symptom onset.

## Background

To maintain functional patient care during the Severe Acute Respiratory Syndrome Coronavirus 2 (SARS-CoV-2) pandemic [1], it is crucial to protect healthcare facilities from nosocomial coronavirus disease 2019 (COVID-19) outbreaks. As such, every hospital needs to separate patients with and without COVID-19 via a reliable triage algorithm to minimize the risk of nosocomial transmission during a time of ever-increasing patient numbers. At our German university hospital, patients with suspected COVID-19 have been triaged using a modified low-dose, computed tomography (CT)-based algorithm, according to Zhang et al. [2]. Due to its rapid action and high detection sensitivity for viral pneumonia, CT has proved highly effective for identifying the majority of COVID-19 cases with a high pre-test probability during the peak of the COVID-19 pandemic [3]. However, a significant limitation of CT imaging is the limited specificity of the imaging findings. Thus, diagnostic uncertainty remains in cases of negative polymerase chain reaction (PCR) testing for SARS-CoV-2. Direct detection of the virus by PCR is only possible for a limited time span and thus may be less sensitive than CT [4]. Thus, in advanced disease stages of COVID-19 PCR may miss a correct diagnosis [5–8]. Moreover, pre-analytics such as the quality of nasopharyngeal swaps also have a significant impact on the sensitivity of PCR assays [9]. Consequently, approximately one-third of patients with distinct COVID-19 CT findings are tested negative for SARS-CoV-2 RNA by PCR rendering correct diagnosis difficult [4].

Infection control management of patients with suspected COVID-19 that cannot be confirmed later on is complex and cost-intensive. To reduce the risk of nosocomial transmission of COVID-19, these patients must be isolated from others and are treated with high personal protection equipment. To correctly diagnose COVID-19 patients, innovative solutions are required.

The main focus of this retrospective study was to evaluate SARS-CoV-2 serology as a supplementary diagnostic method to increase diagnostic accuracy for patients with suspected COVID-19.

## Methods

### Study population

Retrospective evaluation for inclusion in this study was performed for 183 patients with suspected or confirmed COVID-19 admitted to the University Hospital Klinikum rechts der Isar of the Technical University of Munich, or identified during their hospital stay, from the 4^th^ of March to 22^nd^ of April 2020 during the first wave of the COVID-19 pandemic. All patients fulfilled at least one criterion, according to the Wuhan Triage Algorithm (respiratory symptoms/chills plus dyspnea/hypoxia and/or temperature > 37.3 °C and/or absolute lymphocyte counts < 1100/µL) [2], and therefore underwent both low-dose chest CT scanning and SARS-CoV-2 PCR testing of nasopharyngeal swab. Of 183 patients, 30 were excluded: one patient received immunoglobulin therapy due to secondary antibody insufficiency syndrome prior to hospitalization, five patients had incomplete data, and three patients had both negative CT and repeatedly negative PCR testing. As SARS-CoV-2 serology testing had not been implemented into clinical routine at our hospital from the very beginning of the COVID-19 pandemic, 21 patients without SARS-CoV-2 serology test results were excluded. Influenza and respiratory syncytial virus (RSV) co-infection were excluded in all patients using PCR (Cepheid GeneXpert RSV/FLU, California, USA). Patients with positive SARS-CoV-2 PCR ± distinct COVID-19 CT features were defined as confirmed COVID-19 cases (group A), whereas patients with positive CT findings but negative PCR were classified as suspected cases (group B). Every patient underwent a standardized medical history assessment including the documentation of symptom onset prior to hospitalization. If the assessment could not be performed due to cognitive impairment, such as dementia or critical illness (9 patients), the day of hospital admission was defined as the date of symptom onset. Twelve patients first developed clinical symptoms during pre-existing hospitalization.

### Inclusion and exclusion criteria

The following inclusion criteria were defined:

- Age ≥ 17
- Availability of (low-dose) chest CT
- SARS-CoV-2 PCR testing

Patients were excluded from the study in the following cases:

- Plasma separation therapy or immunoglobulin substitution therapy before serological testing
- Negative SARS-CoV-2 PCR and chest CT without COVID-19 suspicious findings
- No SARS-CoV-2 serology available
- Incomplete medical records in terms of the above-listed data criteria

### Ethics statement

The study was approved by the Institutional Ethics Committee of the Klinikum rechts der Isar, Technical University of Munich, which operates according to the Declaration of Helsinki (Approval No. 247/20 S). Due to the retrospective study design, the need for written consent was waived.

### Sample collection

In patients with suspected COVID-19, nasopharyngeal swab samples were collected according to a standardized protocol. On ICU, trachea-bronchial aspirates were alternatively obtained for SARS-COV-2 PCR. To protect our medical staff from COVID-19 transmission, all aerosol producing interventions like bronchoalveolar lavage were avoided if not absolute necessary from a clinical point of view. To economize laboratory capacity and testing efficiency during the pandemic situation, nasopharyngeal and throat swab specimens were pooled. In order to guarantee optimal detection of SARS-CoV-2 by PCR, a standardized swab collection and transport system (Sigma Virocult© & Transwab©, Sigma-Aldrich, St. Louis, Missouri, USA) complying with the CLSI standard M40-A for Quality Control of Specimen Transport Devices was used. Samples were stored at 4 °C until processed. For serological testing, 5–8 mL of blood serum was obtained.

### Laboratory testing

PCR and serological processing were performed at a clinical virology laboratory (Institute of Virology, Technical University of Munich), accredited according to DIN EN ISO 15,189. Ribonucleic acid (RNA) was extracted using the mSample Preparation System DNA kit with standard protocol for the simultaneous extraction of DNA and RNA on a m2000sp device (Abbott, Wiesbaden, Germany). SARS-CoV-2 PCR was performed using "in-house" real-time PCRs on a TaqMan device and primer and probe sets targeting the SARS-CoV-2 N gene according to the protocol of the Center for Disease Control and Prevention (CDC), Atlanta, USA. For screening and quantification the N1 and for confirmation the N3 primer/probe sets were used.

### Validation of SARS-CoV-2 serology

Detection of serum IgM and IgG antibodies for SARS-CoV-2 was performed using a paramagnetic particle chemiluminescent immunoassay (CLIA) on an iFlash 1800 immunoassay analyzer (Shenzhen Yhlo Biotech Co., Shenzhen, China). Specificity of SARS-CoV-2 IgM and IgG serology was validated in-house by analyzing 84 control sera, which were collected during 2019 and stored at −20 °C in our biobank for research purposes. Of 84 control sera, two were positive for SARS-CoV-2 IgG and one for IgM, resulting in an overall specificity of 98%. According to the manufacturer’s specification, sensitivity and specificity of the kits are 97.3% and 96% for IgG, and 86.1% and 99.2% for IgM. In a recently published study [10], sensitivity of 97.6% for IgG and 87.8% for IgM was observed, with an overall specificity of 100%.

### Computed tomography

Study patients underwent a low-dose chest CT using a 256-row scanner (iCT, Philips Healthcare, Best, Netherlands). All CT scans were evaluated for distinct COVID-19 features by a 1st–4th year radiology resident and re-evaluated by an experienced attending radiologist (2–15 years of experience) during routine reporting. CT features were assessed using a standardized in-house classification with five levels of diagnostic certainty: 0 = no signs of COVID-19 features; 1 = infiltration or consolidation not typical for COVID-19 infection; 2 = early stage COVID-19 possible; 3 = typical CT features compatible with early COVID-19; 4 = typical CT features compatible with advanced COVID-19.

### Statistical analysis

The distribution of continuous variables is described by median and range. Categorical data are presented as absolute and relative frequencies. Kaplan–Meier survival curves were chosen to illustrate the time from symptom onset to the event of seroconversion to SARS-CoV-2 serology. Comparison of Kaplan–Meier survival curves was conducted by log-rank testing. Statistical hypothesis testing was performed on two-sided exploratory 0.05* significance levels. All analyses were conducted using IBM SPSS Statistics 20 (version 26, IBM Corp., Armonk, New York, USA) and R (version 3.4.3, R Foundation for Statistical Computing, Vienna, Austria).

## Results

### Baseline characteristics

In total, 153 patients met the criteria for inclusion. The median age of the study population was 68 years (range 17–100). Of the 153 patients, 58 (38%) were female. Median time from symptom onset to hospital admission was 6 days (range 0–32). Of the 153 patients, 53 (35%) required transfer to the intensive care unit (ICU) during hospitalization. In-house mortality was 16% (25/153 patients) at the time of analysis (22^th^ May 2020). According to the study criteria, 107 patients (107/153; 70%) were defined as confirmed COVID-19 (group A: PCR + , CT ±) and 46 patients (46/153; 30%) as suspected COVID-19 (group B: PCR-, CT +). Table 1 shows the difference in baseline characteristics between patient group A and patient group B. Table 2 illustrates the distribution of CT findings between group A and group B according to the level of certainty for COVID-19 using the in-house CT-based COVID-19 classification.

**Table 1:** Difference in baseline characteristics between group A and group B

Baseline characteristics	In total Absolute value (%)	Group A (PCR + ; CT ±) Absolute value (%)	Group B (PCR-; CT +) Absolute value (%)
Number of patients	153 (100)	107 (70)	46 (30)
Age (median)	68	67	69
Sex (male/female)	95/58	70/37	25/21
Comorbidities (Comorbidity Score index points*)			
Coronary artery disease^a^ (1 point)	17 (11)	15 (14)	2 (4)
Heart disease^b^ (1 point)	41 (27)	27 (25)	14 (30)
Cerebrovascular disease^c^ (1 point)	11 (7)	6 (5)	5 (11)
Dementia/Parkinson disease (1 point)	17 (11)	8 (8)	9 (20)
Gastric ulcer disease (1 point)	5 (3)	4 (4)	1 (2)
Chronic pulmonary disease (1 point)	17 (11)	12 (11)	5 (11)
Peripheral vascular disease (1 point)	9 (6)	7 (7)	2 (4)
Mild liver disease^d^ (1 point)	1 (1)	1 (1)	0 (0)
Liver cirrhosis (3 point)	4 (3)	3 (3)	1 (2)
Diabetes without end organ failure (1 point)	22 (14)	18 (17)	4 (9)
Diabetes with end organ failure (2 points)	8 (5)	7 (7)	1 (2)
Renal insufficiency (2 points)	14 (9)	11 (10)	3 (7)
Active tumor disease^e^ (2 points)	11 (7)	9 (8)	2 (4)
Metastatic tumor disease (6 points)	16 (10)	8 (8)	8 (17)
AIDS (6 points)	-	-	-
Comorbidity Score points in total (median/mean)	314 (1/2.05)	209 (1/1.95)	105 (1/2.28)
Median duration of hospitalization in days	16	16	13.5
Number of patients with typical CT findings compatible with a high level of certainty for COVID-19	125 (82)	85 (79)	30 (65)
Deaths	25 (16)	18 (17)	7 (15)
ICU admission	53 (35)	41 (38)	12 (26)
Treatment (Remdesivir)	17	17	0

*Modified comorbidity index referring to Charlson et al. [11].

^a^Including stenting or aortocoronary bypass;^b^including arterial fibrillation, congestive heart failure; ^c^including transient ischemic attack (TIA), stroke; ^d^defined as severe steatosis hepatis; ^e^including solid tumours and haematological malignancies.

**Table 2:** Level of certainty for COVID-19 based on CT findings using in-house radiology classification

Level of certainty for COVID-19	COVID-19 CT classification	Group A (n = 107) abs. no (%)	Group B (n = 46) abs. no (%)
Low	No signs of COVID-19 CT features (category 0)	4 (4)	–
Low	Infiltration or consolidation not typical for COVID-19 (category 1)	–	4 (2)
Low	Early stage of COVID-19 infection possible (category 2)	17 (18)	30 (14)
High	Typical CT features compatible with early COVID-19 (category 3)	23 (25)	20 (9)
High	Typical CT features compatible with advanced COVID-19 (category 4)	56 (60)	46 (21)

### SARS-CoV-2 serology: results

99 of the 153 (65%) patients were SARS-CoV-2 seropositive. Of the seropositive patients, 77% (76/99) showed both IgM and IgG positivity, while 23% (23/99) of patients were only IgG positive. IgM and IgG seroconversions occurred in median 14 days (range 4–32) and 13 days (range 2–32) following SO, respectively.

**Fig. 1:**
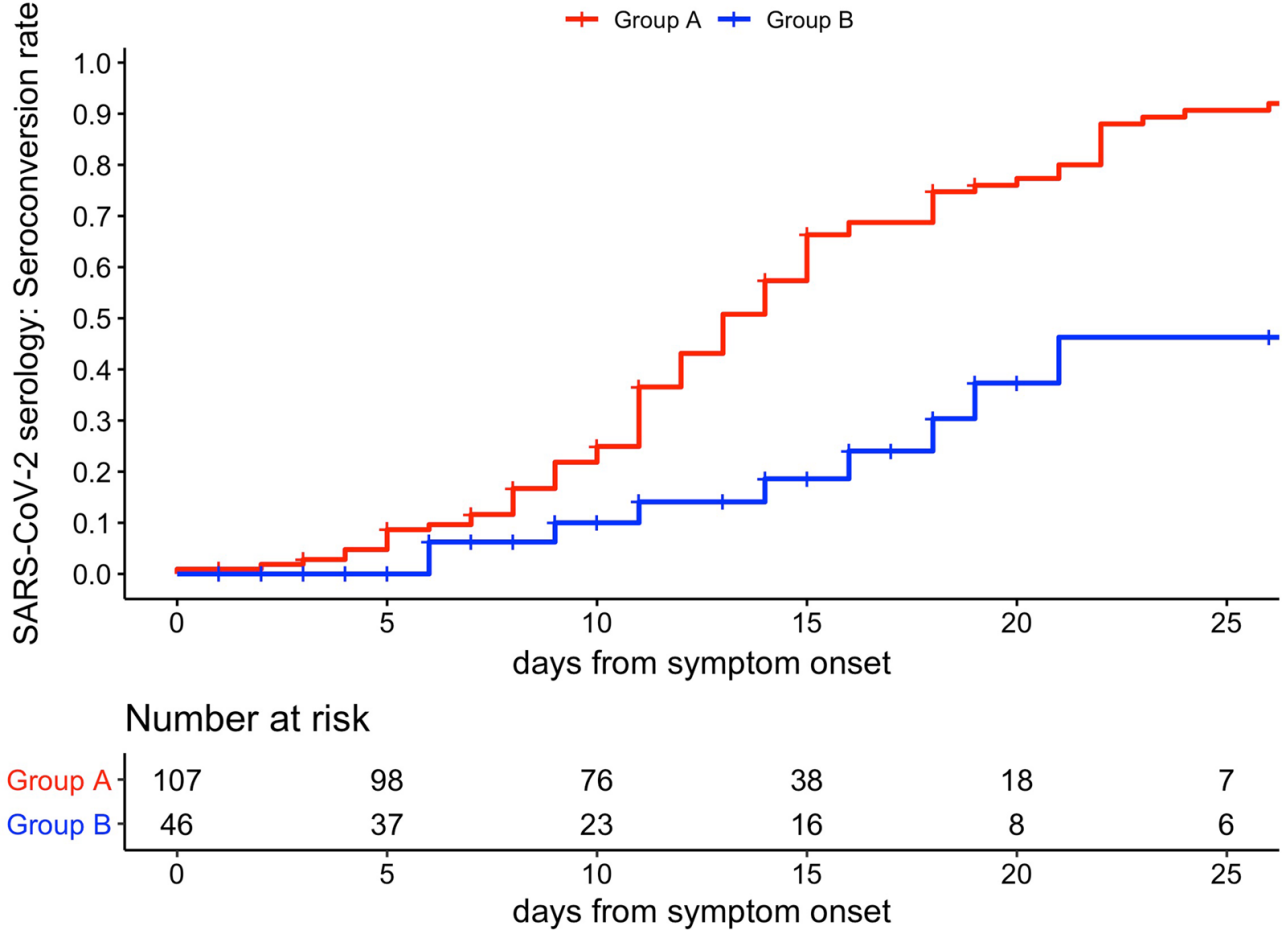
Illustration of seroconversion rates following SO in patients with confirmed COVID-19 (group A, solid red  line) and patients with suspected COVID-19 (group B, solid blue line); p-value < 0.01

Figure 1 displays the difference in seroconversion rates of SARS-CoV-2 serology distinctly for confirmed (group A) and suspected COVID-19 cases (group B). On days 5, 10, 15, 20, and 25 following SO, seroconversion rates of group A and group B were 8%, 25%, 65%, 76%, 91%, and 0%, 10%, 19%, 37% and 46% (p < 0.01), respectively.

**Fig. 2:**
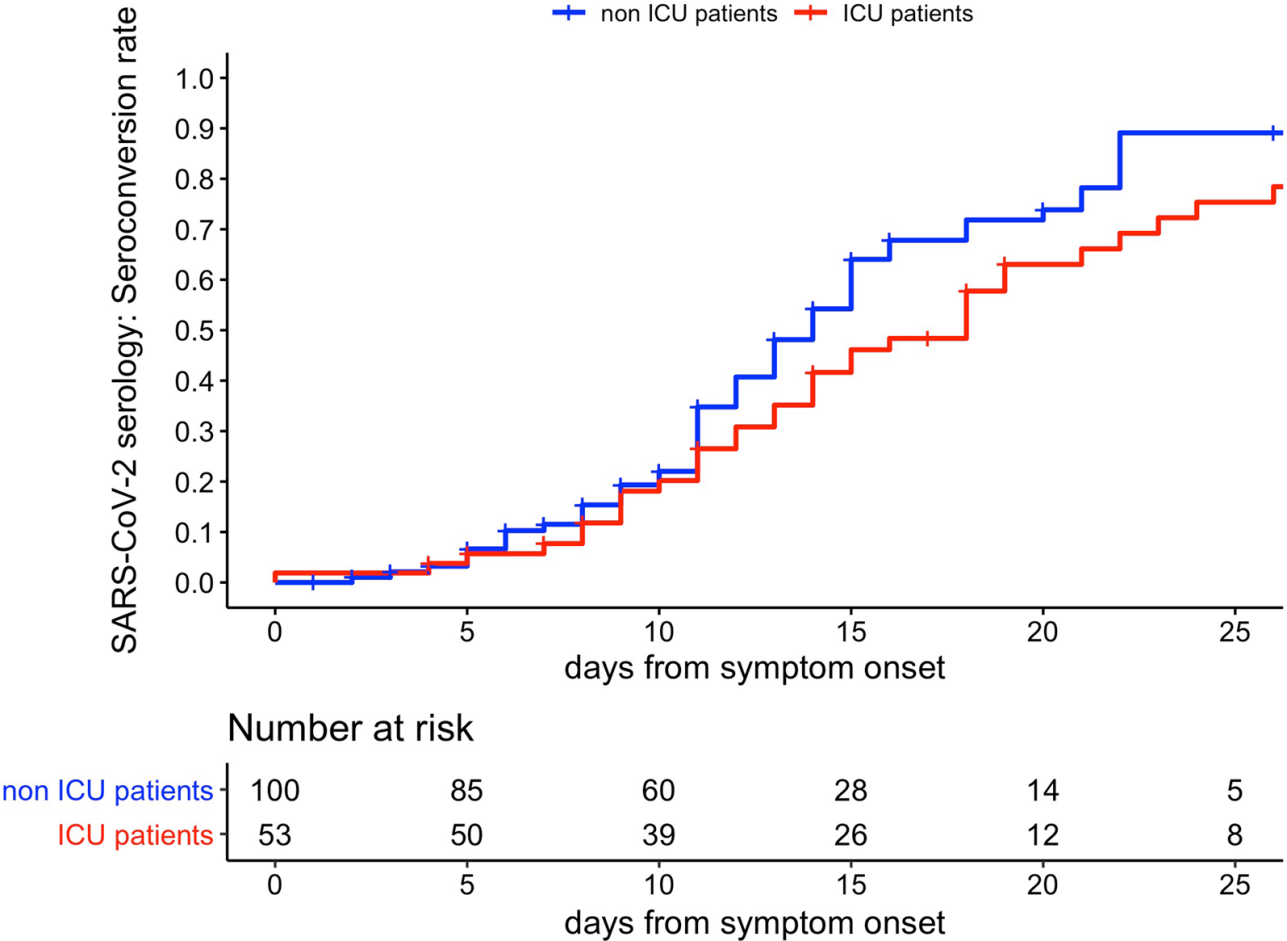
Comparison of seroconversion rates over time between ICU patients (solid red line) and non-ICU patients (solid blue line); p = 0.11

In terms of severity of COVID-19 (Fig. 2), seroconversion was higher and occurred earlier in non-ICU patients than in ICU patients. At 5, 10, 15, 20 and 25 days following SO, seroconversion was 8%, 22%, 64%, 74%, and 89%, respectively, in non-ICU patients, and 6%, 20%, 46%, 63%, 75%, respectively, in ICU patients (p = 0.11).

**Fig. 3:**
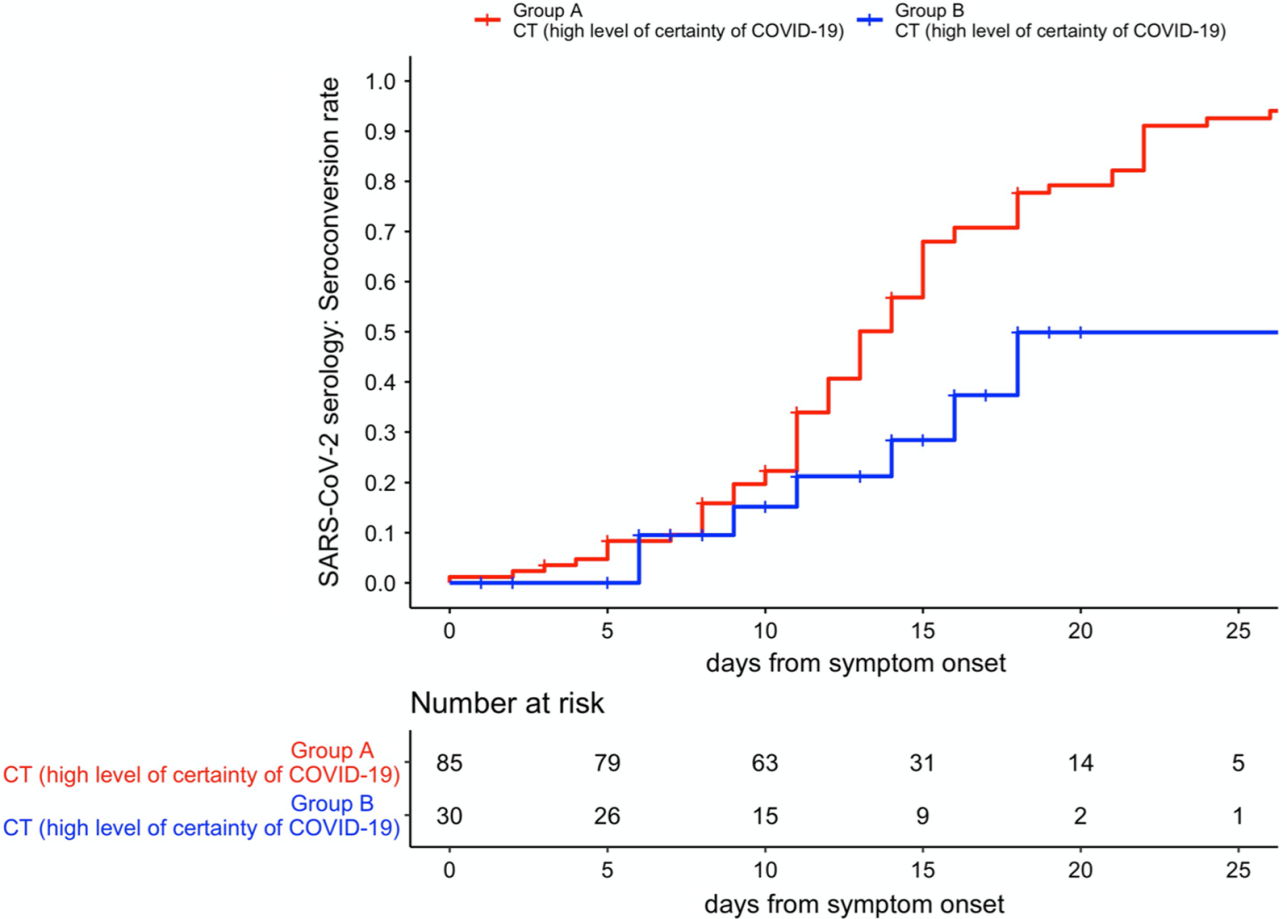
Difference in seroconversion between group A (solid red line) and group B (solid blue line); p < 0.01. Analysis includes only patients with CT findings compatible with a high level of certainty for COVID-19

The impact of CT findings in relation to likelihood of COVID-19 based on seroconversion is demonstrated in Fig. 3. Seroconversion rates of PCR-positive patients (group A) with CT findings compatible with a high level of certainty for COVID-19 (including only category 3 and category 4 according to the in-house CT classification; Table 2) were 8%, 22%, 68%, 79%, and 93% after 5, 10, 15, 20 and 25 days, respectively, following SO. In contrast, seroconversion rates of PCR-negative patients (group B) with CT findings consistent with high level of certainty for COVID-19 (including only category 3 and category 4 according to the in-house CT classification; Table 2) were 0%,15%, 28%, 50%, and 50% at the same time intervals following SO (p < 0.01).

**Fig. 4:**
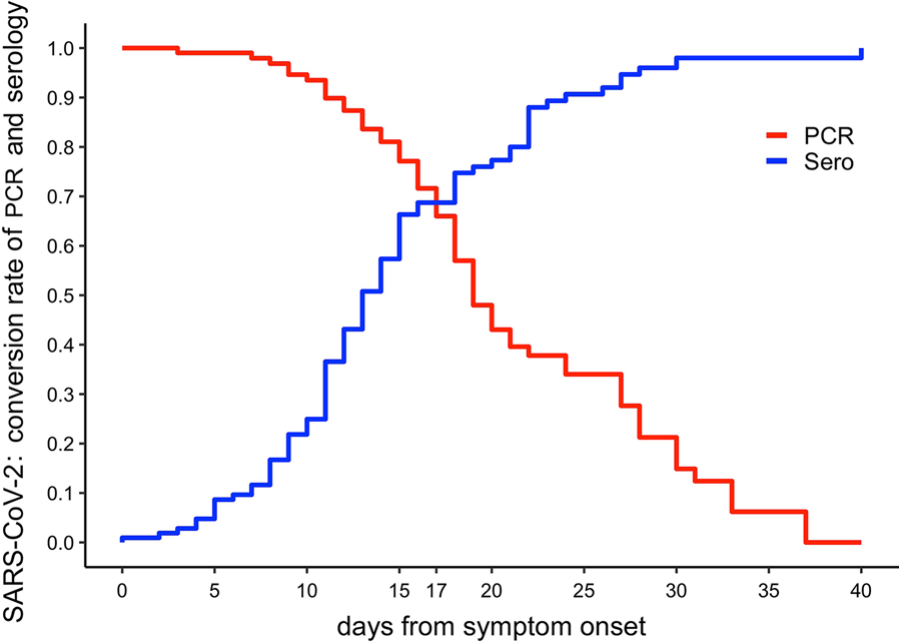
Analysis of conversion rate of SARS-CoV-2 serology and SARS-COV-2 PCR test during hospitalization

Seroconversion and PCR conversion (defined as time point of negativeSARS-CoV-2 PCR) were analyzed during hospitalization (Fig. 4). In the early phase of infection, the detection efficiency of PCR for COVID-19 was higher compared with SARS-CoV-2 serology, whereas detection efficiency of serology for COVID-19 became superior to PCR > 17 days following symptom onset.

In 12 of 46 (26%) suspected cases (group B), positive SARS-CoV-2 serology provided definitive diagnoses of COVID-19. All of these patients underwent at least two SARS-CoV-2 PCR tests (range 2–6). Eight of these 12 patients had typical CT features compatible with a high level of certainty for COVID-19 according to our COVID-19 radiological classification. Based on the findings by Wölfel et al., [6] which showed a COVID-19 seroconversion rate of 100% within 14 days after SO, patients from group A (confirmed COVID-19) and group B (suspected COVID-19) with at least one late serological test > 14 days were separately analyzed in subgroup A1 and subgroup B1. Seroconversion rates of subgroup A1 (confirmed COVID-19) and subgroup B1 (suspected COVID-19) were 92% (59/64 patients) versus 52% (10/19 patients), resulting in a difference of 40%. In subgroup A1, seroconversion did not occur in 8% (5/64) of patients; all five patients suffered from immunosuppressive diseases such as active neoplasia (four patients) or diabetes mellitus (one patient). In comparison, 48% (9/19) of suspected cases with at least one SARS-CoV-2 serology test > 14 days after SO (subgroup B1) had no seroconversion. Retrospective interdisciplinary clinico-radiolocial consensus re-evaluation of these nine patients revealed possible causes other than COVID-19 for their CT findings: one patient underwent bronchoalveolar lavage prior to CT scanning and developed lung edema due to iatrogenic fluid instillation; three patients showed signs of focal lung edema due to cardiac failure; one patient had pulmonary metastatic breast cancer with presumed lymphangiosis carcinomatosis, mimicking COVID-19; one patient`s CT showed signs of breathing artifacts; two patients had CT features in retrospect rather compatible with bacterial pneumonia (lobar pneumonia surrounding by ground-glass opacities with a parapneumonic pleural effusion) as a possible differential diagnosis. For the final one of these nine patients, no other rational differential diagnosis besides COVID-19 seemed to be reasonable.

## Discussion

The CT-based “Wuhan Triage Algorithm” was developed during the peak of the COVID-19 pandemic in Wuhan, China [2]. A core element of this triage algorithm is a low-dose chest-CT, providing faster and more sensitive identification of patients with COVID-19 compared to detection of SARS-CoV-2 RNA by RT-PCR. However, the positive predictive value of highly sensitive diagnostic methods depends heavily on the prevalence of the suspected disease in the tested cohort. In many countries all over the world, nationwide lock-downs and social distancing measures have flattened the curve of the COVID-19 pandemic [12], and consequently the proportion of COVID-19 in patients attending the Emergency Department with fever or respiratory symptoms decreased. This may result in an increase in the numbers of false-positive CT findings for COVID-19. Physicians faced a similar problem regarding diagnosis of other infections such as *Clostridium difficile* or human immunodeficiency virus (HIV) – highly sensitive tests with low positive predictive values due to low prevalence. This problem can be solved by introducing a two-step diagnostic algorithm based on a highly sensitive screening test, followed by a confirmatory test [13]. In this study, SARS-CoV-2 serology was evaluated as a confirmatory test for patients with CT findings suspect of COVID-19. Out of 153 patients, 46 (30%) had a suspected diagnosis of COVID-19, which was based on a combination of clinical findings and CT imaging results. Tao Ai et al. [4] analyzed the correlation of chest CT and PCR testing in COVID-19 in China, revealing a similar proportion of suspected COVID-19 cases with negative PCR but positive CT findings. For the patients in our study with repetitive negative PCR and positive CT findings (group B), positive serology led to the final diagnosis of COVID-19 in 26% (12/46) of cases. As illustrated in the Methods section, the specificity of SARS-CoV-2 serology was found to be high (98%). Nevertheless, differentiation between acute infection and post-infection IgG responses is challenging in the absence of any IgM response. In the current study, in 23% (23/99) of seroconverted patients only IgG was detected. As an increasing seroprevalence of COVID-19 in the population can be expected in the months following the pandemic, SARS-CoV-2 serology may mislead clinicians to diagnose acute COVID-19 in their patients. Therefore, to increase pre-test probability, SARS-CoV-2 serology could be embedded into a two-step diagnostic algorithm using CT as a screening test, followed by SARS-CoV-2 serology and PCR as a confirmation test when acute infection is suspected. This way, a diagnosis of COVID-19 can be confirmed despite repetitive negative PCR testing, if patients present positive SARS-CoV-2 serology and show distinct COVID-19 CT findings.

The sensitivity of SARS-CoV-2 serology increased with time since symptom onset. In PCR-positive patients (group A), SARS-CoV-2 serology achieved a sensitivity of less than 15% within the first week and up to 92% after the third week following SO. Wölfel et al. [6] analyzed seroconversion of patients suffering from COVID-19 based on an assay using cloned spike protein of SARS-COVID-19. In early sera, collected between days three and six, no seroconversion was observed; however, all patients developed an antibody response during monitoring for at least two weeks. These findings clearly illustrate that the use of SARS-CoV-2 serology as a tool to diagnose COVID-19 in the early stage of infection is limited.

In the current study, the median time from symptom onset to hospital admission was six days, which is comparable to other clinical trials [10, 14]. Less than 15% of confirmed cases had a positive antibody response on day six following SO, as shown in Fig. 1. In the early phase of infection (≤ 17 days), the sensitivity of PCR was superior to SARS-CoV-2 serology. However, with prolonged duration of symptoms (> 17 days), PCR became inferior to the detection efficiency of SARS-CoV-2 serology. In comparison with the data from Wölfel et al. observing a seroconversion of all patients within 14 days after symptom onset [6], seroconversion did not occur in all patients and was delayed in the current study, which could be related to the high proportion of severely ill patients. Wölfel et al. [6] analyzed antibody response against COVID-19 in non-severely ill patients during the early containment phase of the COVID-19 pandemic in Germany and, in contrast, 35% of our study patients had to be transferred to the intensive care unit. As shown in Fig. 2, humoral response against COVID-19 in this study tended to occur earlier and more frequently in non-ICU patients than in ICU patients. In contrast to the findings of our study, Fourati et al. [15] observed that all patients admitted to ICU had a positive serology SARS-CoV-2 upon admission. We believe that these discrepant findings can be explained by different test performances of SARS-CoV-2 assays due to the use different antigens. Fourati et al. [15] used an IgG/IgA assay, whereas SARS-CoV-2 antibody detection was conducted by a IgG/IgM assay in the current study. Interestingly, Sun et al. [16] analyzed IgM and IgG responses against SARS-CoV-2 nucleocapsid (N) and spike (S) protein after symptom onset depending on the severity of COVID-19. They found that ICU patients had higher N-IgG than S-IgG than non-ICU patients. Furthermore, S-IgG increased slower than N-IgG in ICU patients after symptom onset. In line with the results of our study, Qu et al. [10] analysed the profile of IgG and IgM antibodies against severe acute respiratory syndrome coronavirus 2 (SARS-CoV-2). IgG and IgM antibodies against SARS-CoV-2 were measured using the same iFlash-SARSCoV-2 IgG/IgM chemiluminescent immunoassay kit (C86095G/C86095M, YHLO BIOTECH, Shenzhen) as we did in our study. Compared to patients with mild or moderate COVID-19, they also observed delayed IgG and IgM antibody responses in critical ill patients suffering from COVID-19.

Interestingly, median seroconversion of IgG was observed slightly earlier than median seroconversion of IgM. This could be related to a well-known technical problem with serological assays: avidity of IgG antibodies is higher compared to that of IgM antibodies, and so IgG can outcompete IgM for viral epitopes. In addition, numbers are still low. Thus, this phenomenon requires further analysis.

The difference in seroconversion rates between PCR-positive and PCR-negative patients highlights the intermediate specificity of CT imaging findings for COVID19 pneumonia. At day 25 after symptom onset, seroconversion occurred only in up to 46% of PCR-negative patients, compared with 91% of PCR-positive patients (Fig. 1). Viral infections such as RSV, influenza, or human metapneumovirus [17] along with several non-infectious causes, can present similar CT changes such as multifocal consolidation or ground-glass opacities in the lungs. Diagnosis of COVID-19 in all group B patients with negative SARS-CoV-2 serology > 14 d after SO (9/19 patients) was retrospectively re-evaluated by an interdisciplinary Senior Consultant Physician Team from the Department of Radiology and the Department of Internal Medicine and Infectious Diseases. Only one patient out of nine had a differential diagnosis workup which revealed no other possible cause for the CT findings. Most of the remaining patients (4/9) suffered from pre-existing cardiovascular diseases and showed radiological signs of pulmonary edema such as septal lines or peribronchial cuffing. In contrast to characteristic lobular pneumonia, CT findings for COVID-19 are often diffuse and bilaterally localized, and therefore sometimes difficult to distinguish from a cardiogenic pulmonary edema. Pulmonary edema can also be caused by the underlying inflammatory process of pneumonia, which further complicates the distinction between COVID-19 and cardiogenic pulmonary edema. Clinical and radiological re-evaluation based on response to diuretic therapy may be the best way to differentiate a cardiogenic pulmonary lung edema from COVID-related CT findings. The CT of one patient with a pre-existing cardiovascular disease was initially rated as highly suspicious for COVID-19; however, seven days after the initiation of diuretic therapy, another CT scan was performed which no longer showed signs of typical COVID-19 related findings. From this experience it can be concluded that diagnosis of COVID-19 based solely on CT-findings is not highly reliable despite the high prevalence of COVID-19 in the current pandemic and therefore should either be confirmed by SARS-CoV-2 PCR or SARS-CoV-2 serology.

One limitation of this study is that the time points of serological testing were not standardized due to its retrospective design, which represents a potential bias to the time of seroconversion. Nevertheless, this potential bias does not appear to have impacted upon the major finding of the study, namely that a significant proportion of typical COVID-19 CT findings could be false positive.

## Conclusion

Pre-test probability of SARS-CoV-2 serology in patients with typical COVID-19 CT findings can be considered sufficient to confirm COVID-19 despite the absence of a positive PCR. Nonetheless, negative SARS-CoV-2 serology does not exclude COVID-19. As the detection probability of antibody response increases with duration from symptom onset, the repetition of serological testing seems to be reasonable for PCR-negative patients who have recently developed symptoms. Finally, diagnosis of COVID-19 should be questioned in cases where patients have repetitive negative PCR and serological testing, despite distinct COVID-19 CT features.

## Data Availability

The datasets used and/or analysed during the current study are available from the corresponding author on reasonable request.

## Abbreviations

TIA: Transient ischemic attack
SR: Seroconversion rates
SO: Symptom onset
COVID-19: Coronavirus disease 2019
CT: Computed tomography
PCR: Polymerase chain reaction
d: Day
SARS-CoV-2: Severe Acute Respiratory Syndrome Coronavirus 2
RSV: Respiratory syncytial virus
CLIA: Chemiluminescent immunoassay
